# Bridging the Gap: A Mixed-Methods Evaluation of a New Rural Maternity Care Center Amid Nationwide Closures

**DOI:** 10.3390/ijerph23010102

**Published:** 2026-01-12

**Authors:** Kathryn Wouk, Ellen Chetwynd, Emily C. Sheffield, Marni Gwyther Holder, Kelly Holder, Isabella C. A. Higgins, Moriah Barker, Tim Smith, Breanna van Heerden, Dana Iglesias, Andrea Dotson, Margaret Helton

**Affiliations:** 1 Pacific Institute for Research and Evaluation, Beltsville, MD 20705, USA; 2 Gillings School of Global Public Health, University of North Carolina, Chapel Hill, NC 27514, USA; ihiggins@email.unc.edu (I.C.A.H.); brejohn@email.unc.edu (B.v.H.); 3 Department of Family Medicine, University of North Carolina, Chapel Hill, NC 27514, USA; ellen_chetwynd@med.unc.edu (E.C.); marni_holder@med.unc.edu (M.G.H.); kholder@campbell.edu (K.H.); tismith@email.unc.edu (T.S.); dana_iglesias@med.unc.edu (D.I.); margaret_helton@med.unc.edu (M.H.); 4 Division of Health Policy and Management, School of Public Health, University of Minnesota, Minneapolis, MN 55455, USA; sheff074@umn.edu; 5 School of Osteopathic Medicine, Campbell University, Lillington, NC 27546, USA; 6 Department of Family & Community Medicine, Wake Forest University, Winston-Salem, NC 27101, USA; moriah_barker@med.unc.edu; 7 Department of Family Medicine and Community Health, Duke University, Durham, NC 27705, USA; andrea.dotson@duke.edu

**Keywords:** rural, maternity care, family medicine, birth, mixed methods

## Abstract

**Highlights:**

**Public health relevance—How does this work relate to a public health issue?**

- Rural maternity unit closures exacerbate geographic and racial/ethnic inequities in maternal and neonatal health outcomes; this study examines the impact of restoring local access to maternity care.
- By evaluating both clinical outcomes and patient experiences after the reopening of a rural maternity care center, this work addresses a critical gap in understanding how service restoration affects access, quality, and patient-centered care.

**Public health significance—Why is this work of significance to public health?**

- Findings demonstrate that reopening a rural Level I Maternity Care Center can maintain safe and comparable labor and delivery outcomes while reducing travel burden by half, thereby improving access to timely, equitable perinatal care.
- The study highlights a resource-appropriate maternity care unit of family physicians and midwives that provides a model for delivering quality rural maternity services in underserved regions nationally.

**Public health implications—What are the key implications or messages for practitioners, policy makers and/or researchers in public health?**

- Researchers and practitioners should explore scalable, multidisciplinary staffing models and community-informed implementation strategies to expand high-quality rural perinatal care where maternity care services have been lost.
- To sustain rural maternity care, health systems and policymakers must invest resources to increase reimbursement for these services, incentivize practice in rural areas, and ensure ongoing training and interprofessional development for rural maternity and newborn teams.

**Abstract:**

The closure of rural maternity units in hospitals across the United States contributes to health inequities; however, little is known about the effects of reopening maternity services in this context. We conducted a mixed-methods study to characterize labor and delivery outcomes and patient experiences associated with the reopening of a rural Level 1 Maternity Care Center (MCC) at a critical access hospital. We compared clinical outcomes and distance to care for patients who gave birth at the rural MCC in the three years after its opening with outcomes from a similar low-risk and geographically located sample who gave birth at a large suburban academic medical center in the same hospital system in the three years before the MCC reopened. We also conducted in-depth interviews with patients who gave birth at the MCC. Labor and delivery outcomes were similar across both groups, with significantly more care provided by family physicians and midwives and lower neonatal intensive care unit use at the MCC. The opening of the MCC halved the distance patients traveled to give birth, and patients reported high rates of satisfaction. Rural maternity care centers can improve access to quality care closer to home using a resource-appropriate model.

## 1. Introduction

There is a maternity care crisis in the United States (US), as increases in obstetric unit closures over the past few decades have created large geographic areas without access to pregnancy- and birth-related care. Rural communities are more adversely affected, facing significant losses of maternity services for prenatal care and childbirth [1]. From 2014 to 2018, more than half of rural counties lacked hospital-based maternity care, and an additional 53 rural counties lost services during this time period [2]. The percent of rural hospitals without hospital-based maternity care increased from 43% in 2010 to 52% in 2022 [3], and by 2022, over two-thirds of rural hospitals in eight states were without these services [4]. Financial concerns complicate rural hospitals’ ability to maintain low-volume obstetric units, as the costs of obstetric services can be difficult to offset with revenue amid low patient volumes and low reimbursement [5]. Hospital systems aim to reduce costs by closing low-volume rural maternity units and centralizing services; however, these closures increase distance to maternity care by an average of 29 miles [6], and are associated with increases in elective early induction and low-risk cesarean births [7,8,9,10], non-hospital births [8,11,12], preterm births [8,12,13], admission to neonatal intensive care units (NICUs) [9], and neonatal and maternal morbidity [8,13]. The closure of maternity units also contributes to a loss of local prenatal care providers servicing rural communities, increasing the risk that pregnant patients will experience medical problems that remain undetected or untreated until labor begins [14,15].

Patients affected by rural maternity care center closures in the US are disproportionately uninsured or publicly insured, low-income, and from minoritized racial and ethnic groups. Medicaid finances over half of all births to rural residents, and rural populations are more likely than their urban counterparts to be uninsured before, during, and after birth [16,17]. As Medicaid typically reimburses less for birth care services than private insurers, rural health insurance coverage inequities drive inadequate reimbursement rates for rural maternity providers and difficulties generating revenue from obstetric services for low-volume rural hospitals [5]. Communities with higher proportions of reproductive-age Black and Hispanic women also more often lack maternity care access [6,8]. Low-income patients with fewer resources are more likely to have pregnancies complicated by social determinants that increase health risks, such as high blood pressure and low birthweight, and to be negatively impacted by increased distances to maternity care [8,18].

The US South has experienced a disproportionate share of rural maternity care center closures [19]. Between 2010 and 2022, 8 rural hospitals lost maternity services in North Carolina (NC) [4], a state with a substantial payment gap between Medicaid and private payer reimbursement for labor and delivery care [20]. As of 2022, 44% of rural hospitals in NC lacked obstetric services, compared to 27% of urban NC hospitals [4]. The increased distance to care caused by maternity unit closures is associated with higher levels of emotional and financial stress among rural pregnant patients in Western NC [21].

In September 2020, a critical access hospital (CAH) that is part of a state-funded health system in NC reopened maternity services after a 30-year closure. A CAH is a rural health care facility with up to 25 beds, designated by the Centers for Medicare & Medicaid Services (CMS) to provide essential services in isolated communities at a significant distance from other hospitals [22]. The rural Level 1 Maternity Care Center (MCC) aims to improve local access by providing labor and delivery services to pregnant patients and newborns, reducing distance to care. Despite a growing literature on the adverse access and birth outcomes that may follow rural maternity care closures, rigorous evidence on the health and patient experience impacts of opening or reopening rural hospital maternity services have not yet been documented.

To address this gap, we used a mixed-methods approach that integrates clinical outcomes with patient experiences, providing a comprehensive assessment of the safety and acceptability of restoring maternity services in a rural hospital. This mixed-methods analysis compares labor and delivery outcomes for patients giving birth in the newly opened MCC with those of a similar cohort who gave birth at the suburban tertiary care facility that served this population in the three years prior to the MCC opening. We interpret the quantitative data alongside qualitative findings from in-depth interviews with people who gave birth at the rural MCC to complement clinical outcomes with patient-centered experiences of care.

## 2. Materials and Methods

### 2.1. Setting and Design

We collected data from patients of the rural MCC that opened in September of 2020 in a small NC town with nearly 8000 residents that is considered a “small town core” under the Rural-Urban Commuting Area Codes [23]. The MCC is staffed by family medicine physicians (both with and without surgical cesarean training), certified nurse-midwives (CNMs), and one part-time obstetrician. Anesthesia services were initially contracted from the suburban academic center, before engaging a private anesthesia vendor service experienced in providing coverage in rural settings, and primarily utilizing a certified registered nurse anesthetist (CRNA) staffing model. A Level 1 newborn nursery was implemented with the MCC, with neonates requiring a higher level of care transferred by ambulance or helicopter to the closest available hospital, most commonly to the academic center in the same system with a Level IV neonatal intensive care unit.

Prior to the opening of the MCC, this rural population was primarily served by a large suburban academic medical center in the same hospital system that attended almost 4500 births annually. We compared quantitative patient data from the MCC with patient data from this suburban tertiary care facility in the three years prior to the MCC opening. The academic medical center trains both obstetrics and family medicine residents, while the MCC infrequently had trainees involved in care in its first two years.

We used a parallel mixed-methods design in which quantitative and qualitative data were collected simultaneously and analyzed separately [24]. This approach was selected because both data sources were generated as part of the same three-year evaluation period and reflected overlapping phases of service delivery. This parallel design allowed the evaluation team to examine clinical outcomes and patient experiences within the same policy context and time frame instead of privileging one method of data collection to subsequently inform or follow the other. The quantitative data provide information on clinical outcomes, while the qualitative data provide contextual information on experiences of care from the patient perspective. We combined the results from these two data sets to provide a comprehensive picture of the effect on patient outcomes of opening and sustaining a rural MCC amidst a national landscape of rural maternity closures. This study was approved by the Institutional Review Board of the University of North Carolina at Chapel Hill (IRB #21-0386, IRB #20-2115, IRB#21-1147).

### 2.2. Quantitative Data Collection

We used electronic health records (EHR) to extract demographic and clinical data for patients who delivered at the MCC and a similar cohort of patients who delivered at the suburban tertiary care facility serving the same geographic population in the three years prior to the MCC opening. The MCC cohort comprised all 402 patients who delivered on the unit between its opening on 9 September 2020, until 8 September 2023. The comparison group comprised patients who delivered at the suburban facility between 1 January 2017, and 31 December 2019, lived in the MCC catchment area, and met the low-risk criteria for admission to the MCC.

**Figure 1:**
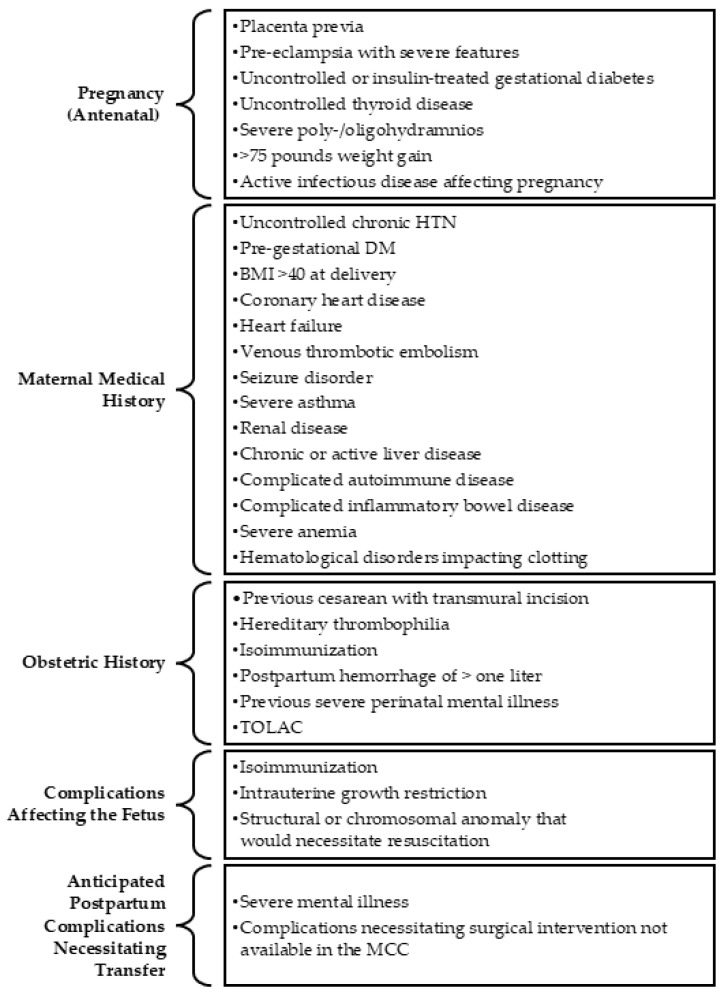
Exclusion Criteria for Level 1 Maternity Care Center. Note. HTN = hypertension; DM = diabetes mellitus; BMI = Body Mass Index; TOLAC = trial of labor after cesarean; MCC = Maternity Care Center.

**Figure 2:**
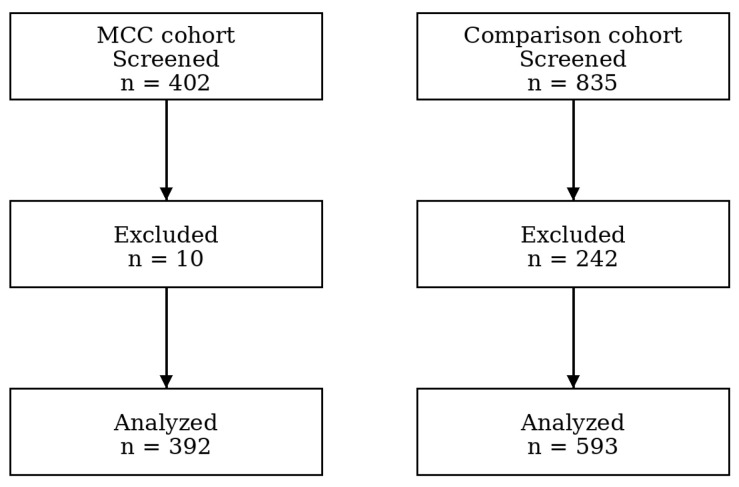
Flow diagram for quantitative analysis.

The MCC catchment area was defined using the patient panels of five proximate prenatal clinics in the same county whose patients delivered at the MCC after its opening, including the county health department, a federally qualified health center with two locations in the county, and two local family medicine clinics affiliated with the hospital system. The data for the comparison cohort was provided by the Perinatal Research Service Center (PRSC) at the School of Medicine at the suburban tertiary care facility. The PRSC used Structured Query Language to extract data from the facility’s health data warehouse, and then systematically derived information from the EHR using clinical business rules approved by facility clinicians which guarantees that the data elements in the integrated data set have data lineage back to Epic’s Caboodle and Clarity databases. After receiving these data on 835 unique deliveries, the research team used ICD-10 codes to define the risk status of each patient on admission to labor and delivery using the exclusion criteria adopted by the Level 1 (basic care to people with low-risk pregnancies) MCC at its opening. We conducted an EHR chart review to exclude patients who would not have met the criteria for admission as a low-risk delivery to the MCC had the unit been available at the time (Figure 1). Of the 835 patients, 242 were excluded from the comparison sample using these inclusion and exclusion criteria. These criteria were also used post hoc to exclude patients who delivered at the MCC emergently, but who did not meet the criteria for delivery at a Level 1 MCC (e.g., breech presentation or preterm delivery <37 weeks’ gestation). Of the 402 MCC patients described in Table 1, 10 were excluded from the quantitative outcome analysis because they would not have met the criteria for a Level 1 delivery (Figure 2).

For both MCC and comparison cohorts, investigators (KW, MGH, MB, BJ, TS, or KH) extracted data on patient demographics, maternal conditions during pregnancy, delivery characteristics, and birth outcomes from the EHR. The patient’s home address as listed in the EHR was extracted to calculate distance to care. Double coding and random audits maintained the quality of abstracted data. Primary labor and delivery outcomes of interest were defined as the following: labor type (spontaneous, induced, scheduled C-section); delivery service type (obstetrics/gynecology, family medicine, or certified nurse-midwifery); delivery method (vaginal, primary C-section, or repeat C-section); epidural use among vaginal deliveries (yes/no); birth outcome (live birth, intrauterine fetal demise (IUFD)); postpartum hemorrhage (>1000 mL blood loss); gestational age at birth (preterm, defined as less than 37 completed weeks’ gestation; or term defined as 37 completed weeks’ gestation or more); birthweight (low, defined as less than 2500 g; normal, defined as 2500–3999 g; or large for gestational age, defined as 4000 g or more); Apgar scores at five minutes (0–10); neonatal intensive care unit stay (yes/no); and infant feeding status at discharge (any breastfeeding, only formula feeding, or not applicable due to IUFD/neonatal death). As this analysis comprises unique deliveries meeting inclusion criteria, a few patients delivering more than once during the years of this study may be represented multiple times in a single cohort or across MCC and comparison cohorts at different points in time. Significance was set at *p* < 0.05.

### 2.3. Qualitative Data Collection

In-depth telephone interviews were conducted with English- or Spanish-speaking patients who gave birth at the MCC during the first year of operation. Patients were contacted at least two weeks and up to ten months after the delivery and were excluded if their chart indicated an infant death, if the EHR was protected, or if they had an emergent delivery at the MCC but would otherwise not have met the Level 1 criteria. From a list of 84 eligible patients, 13 were recruited and completed an approximately half-hour-long phone interview between June and July of 2021. Recruitment continued alongside the process of iterative data analysis until saturation was reached, determined as the point at which new themes were no longer being identified in the data.

A structured interview guide was developed based on a review of the literature and with particular attention to the Birth Satisfaction Scale [25]. Participants were recruited by phone, verbally consented, and then interviewed in English (ES) or Spanish (a native Spanish-speaking graduate research intern). Interviews were recorded, transcribed, and linked with patient hospital records. Participants received gift cards once interviews were completed. Interviews were transcribed verbatim using Sonix transcription software (v1) [26]. Spanish interviews were translated into English by the software and the Spanish translation was verified by a Spanish-speaking researcher. Both English and Spanish transcriptions were checked against audio recordings and corrected before analysis (ES and IH).

### 2.4. Data Analysis

Descriptive statistics were calculated for the full quantitative sample using SAS 9.4 (SAS Institute Inc., Cary, NC, USA). Frequencies and percentages were used to describe categorical characteristics, while means and standard deviations were used for continuous variables.

We assessed whether labor and delivery outcomes differed between patients who delivered at the MCC compared to those who delivered at the suburban tertiary care center. We did not use quantitative balancing for indicators of social risk because the comparison cohort was drawn from the same source prenatal clinics as MCC patients and was therefore expected to be representative of the underlying population of interest. Binary outcomes (e.g., birth outcome) were modeled using logistic regression, while multilevel outcomes (e.g., labor type) were modeled using multinomial logistic regression (generalized logit link), estimating separate log-odds for each category compared to the referent. We report odds ratios (ORs) and 95% confidence intervals (95% CIs) comparing the suburban to the rural hospital setting, along with two-sided *p*-values from Wald chi-square tests. Continuous outcomes (e.g., Apgar scores) were compared between hospitals using two-sample t tests with unequal variances, reporting Satterthwaite *p*-values. All models were unadjusted, and missing data were handled using complete-case analysis (listwise deletion). Analyses were conducted using SAS, version 9.4 (SAS Institute Inc., Cary, NC, USA).

We derived a variable for distance to care by calculating travel distance in miles to the MCC for the cohort of patients who delivered at the MCC using their home address. For the MCC cohort, we geocoded the recorded home address at delivery to latitude/longitude coordinates; two MCC records listed PO box addresses and were therefore assigned maternal location at the ZIP-code centroid. There were no missing data. For the comparison cohort, the PRSC provided a pre-calculated distance using ZIP-code centroids and the geocoded latitude/longitude of the delivery hospital for all patients; therefore, there were no PO box issues. Both distance variables were created using geocoding tools in Stata 17 (StataCorp, College Station, TX, USA). As a sensitivity analysis, we recalculated MCC distances using maternal ZIP-code centroids to harmonize spatial resolution with the comparison cohort; results were substantively similar. Therefore, we present the original comparison here. Once distance to care was calculated, home addresses were removed from the dataset.

Patient interviews were reviewed and coded using Taguette software (v1.2.0) [27] based on response elements. Three researchers (EC, ES, and IH) inductively coded the interviews. A deductive codebook was produced by a single author (ES) as a reference used by the second two researchers, each of whom coded half of the transcripts. All three researchers coded the same set of three interviews to co-develop an inductive codebook for the interviews; these additional inductive codes were agreed upon and defined by consensus among all three researchers and the codebook was updated. Next, the group of interviews was coded iteratively by each researcher acting as a primary, secondary, and tiebreaking coder for each third of the interviews. When a primary and secondary coder disagreed on a definition or code for an interview, the tiebreaking coder reviewed the disagreement and came to a final decision. Conversation continued until consensus was reached on all definitions and codes. Two researchers (ES and IH) reviewed the finalized codes separately and agreed on final themes identified in the full set of interviews.

Data integration occurred during the interpretation phase of analysis, after quantitative and qualitative findings had been analyzed independently. Members of the evaluation team jointly reviewed results from both data sources and systematically compared qualitative themes with quantitative outcomes using a side-by-side approach. We examined areas of convergence, complementarity, and divergence, and we developed a joint display that visually merged qualitative themes with relevant quantitative findings. Because the qualitative interview guides were developed independently of quantitative findings and analyses were conducted concurrently, integration was interpretive rather than iterative, allowing each data source to retain analytic independence while contributing to a comprehensive understanding of patient outcomes and experiences.

## 3. Results

### 3.1. Quantitative Findings

#### 3.1.1. Descriptive Characteristics

During its first three years of service, 402 patients gave birth at the MCC (Table 1). This sample was almost 70% Hispanic/Latino and 42% spoke Spanish as their preferred language. Most were insured by Medicaid (69%) or were uninsured (17%). Maternal age ranged from 16 to 44 years with a mean age of 27 years. Almost 65% of the sample was multiparous and over 90% had no history of preterm birth (Table 1).

There were 593 patients from the MCC catchment area who would have met the low-risk criteria for admission but delivered at the suburban tertiary care facility in the three years prior to the MCC opening. This sample did not differ significantly from the MCC sample on race/ethnicity, preferred language, maternal age, parity, or history of preterm delivery. However, the comparison sample differed significantly by insurance status at delivery, with a higher proportion of Medicaid-insured compared with uninsured or privately insured deliveries (Table 1).

#### 3.1.2. Labor and Delivery Outcomes

Statistical comparisons of labor and delivery outcomes between the cohort that delivered at the MCC versus the suburban hospital showed no significant differences in labor or delivery type, epidural use, birth outcome, postpartum hemorrhage, gestational age at birth, birthweight, or infant feeding status at discharge (Table 2). Only about 2% of infants in both cohorts were born at a low birthweight and mean five-minute Apgar scores were similar across both cohorts, indicating good condition in the minutes after birth [28].

Significant differences (*p* < 0.05) between the MCC and comparison cohorts were observed for the delivery service type, reflecting the different hospitals’ staffing, as well as for NICU stays (Table 2). Deliveries at the MCC were significantly more likely to be attended by a family medicine physician (68.1%) or certified nurse-midwife (30.4%), with only 1.5% attended by an obstetrician, while deliveries at the suburban tertiary care center were more likely to be attended by an obstetrician (84.1%) (*p* < 0.0001). Infants at the suburban center were significantly more likely to experience a NICU stay after delivery (33.6%) compared with infants at the MCC (2.5%) who were transferred as necessary to the closest NICU (*p* < 0.0001) shortly after birth (Table 2).

#### 3.1.3. Distance to Care

**Figure 3:**
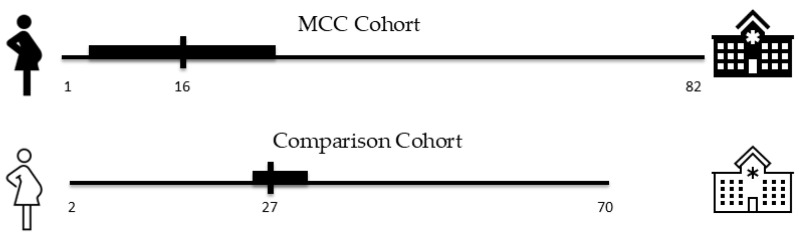
Distance to delivery care (miles).

Distance to delivery care ranged from 1 to 82 miles for the MCC cohort, although approximately one-third of patients (32%) lived 5 or fewer miles from the hospital. Distance to delivery care ranged from 2 to 70 miles from the tertiary care hospital, with less than 1% of patients residing 5 or fewer miles from the hospital. Median distance to care was 16 miles for the MCC cohort and 27 miles for the comparison cohort. Therefore, median distance to delivery care was nearly halved after the availability of the MCC (Figure 3).

### 3.2. Qualitative Findings

#### 3.2.1. Descriptive Characteristics

In the subsample of 13 MCC patients interviewed for the qualitative study, eight identified as Hispanic, three as non-Hispanic white, and two as non-Hispanic Black. All but one were multiparous (92.3%). Nine (61.2%) were insured with Medicaid. Eight interviews were completed in English (62%) and five in Spanish (38%). Almost 85% of the sample reported having a vaginal delivery, and three people reported an induction versus spontaneous labor. All interview participants were breastfeeding at discharge, including eight (61.5%) who were exclusively breastfeeding.

#### 3.2.2. Thematic Findings

Themes identified in the qualitative interviews included: (1) distance to care; (2) provider model and hospital care environment; (3) patient agency/decision-making; and (4) perception changes and recommendations.

Distance to Care was a salient factor for many MCC patients. Distance to care improved access and relieved worry for most participants (69%, *n* = 9). Respondent 4 described, “I had my baby within 15 min of arriving at the hospital. So, I think that if [the MCC] had not been there, I would have had my baby in a car.” Respondent 12 was happy with her hospital choice, stating “I did not want to consider traveling the 45 min it normally took me to get to the other location.” For some, the shorter distance alleviated childcare concerns: Respondent 9 noted, “I have a child that suffers from [medical condition], and I didn’t want to go very far to the other hospital…That was the most important thing, to not go so far away because of my son.” Respondent 1 discussed the benefit of proximity to the hospital in relation to caring for an older child:

“So, my husband and I spoke and one of the advantages that we looked at the most was that it was here near the house and since we have two; I have a little girl of seven years old. He told me, we are close, I can come and go from the hospital to see the girl. And that was also what motivated us the most in the child being born here.”

Respondent 13 noted that distance was the main reason she decided to give birth at the MCC, adding, “And, yeah, since my pregnancy wasn’t a high risk, you know, I would just go to a closer place to where I live.”

The Provider Model and Hospital Care Environment of the MCC were mentioned by many interviewees as impacting their birth experiences. Staff demeanors were described positively, such as “super accommodating” (Respondent 4), “professional” (Respondent 10), “friendly” (Respondents 1, 3, 7, 8, 11), “kind” (Respondents 1, 3, 9), “encouraging”, (Respondent 4, 11, 13), and “very present, but not overbearing” (Respondent 11). Overall attentiveness was described by Respondent 6: “I’d seen [doctor’s name] the whole time I was there. I mean, usually you see the doctor one time, and you never see him again.” Respondent 6 added that small gestures made the experience more personal, noting that the doctor “played her music for me when I was laboring, she was like, you like music? And I was like, yeah. So, she put on some different music to help me through it. It was like the time, like if something was wrong, somebody was there.” Respondent 10 noted that the nurses “…were kinda like friends, you know? They wanted to be there, they enjoyed their job, they cared about my baby. It was amazing.” Respondent 8 said, “I don’t know if the nurses there was mamas too, but they make you feel really comfortable. I just don’t know how to explain it, they were great.”

The MCC staff facilitated the involvement of birthing patients’ family members or other support people in the births. Respondent 1 described how staff engaged her husband: “My husband, he had always wanted to have the experience of cutting the umbilical cord…it was going to be a Caesarean section. He said, ‘ok well now I’m not going to be able to do it,’ and they gave him the opportunity to do it. Everything was very, very special.”

Several patients commented on the physical environment of the MCC. Respondent 10 mentioned the small size of the hospital: “I liked that it was small, because big [hospitals] are so like robotic and stoic.” Respondent 6 described the ease of accessing the smaller local hospital, noting, “I was able actually to park and actually walk into the hospital versus having to be dropped off, have to wait.” Respondent 11 appreciated the larger size of birthing rooms: “The rooms were bigger, the showers were bigger for afterwards, which was a lot of help because with my first two, the showers were so tiny it was hard to move after giving birth.”

Most patients in our qualitative sample did not consider the lack of a NICU or other specialized care unit when selecting the MCC as their delivery hospital (*n* = 10). This may have been less important to a predominantly multiparous and low-risk sample, as Respondent 12 explained, “I knew I was low-risk, I was not a high-risk pregnancy. Pretty standard on my prior deliveries with no complications and pretty standard textbook delivery.” However, Respondent 4 said that the lack of a NICU “was one of my big concerns” when deciding where to give birth. However, she also noted feeling comforted that MCC providers had transfer guidelines and working relationships with obstetricians at the suburban tertiary care facility where the closest NICU was available, adding “And so they would get me or my baby there if they had to.” Two patients hoped that the MCC would get a NICU in the future. Respondent 8, who had an emergency cesarean section at the MCC, said “I mean, anything to do to help [the MCC] for their maternity ward, I would do it, just so they can end up getting the NICU for mothers like me who need it.” Respondent 11 also mentioned, “I really don’t have any complaints. In the future, I would like to see them have a NICU, just in case. But other than that, they were wonderful.”

Patient Agency/Decision-making were common themes regarding patients’ experiences giving birth at the MCC. Patients in the qualitative sample overwhelmingly discussed experiences in which MCC providers fostered shared decision-making during their care. Respondent 11 described offers of pain management as being non-suggestive: “They were very like, ‘whatever you want—if you want medicine, that’s fine, if you don’t, then that’s fine, too. We’ll talk you through all of it.’ Never pushy on either one, which was fantastic because sometimes I felt that way at the other place [where I previously gave birth].” Nonmedical pain management options offered include using the bathtub, birthing ball, rebozo, and music. Respondent 2 said, “I was really happy because they really listened to my birth plan and what I wanted to do. And offered different options, like getting on the birthing ball.” Respondent 6 described feeling in more control at the MCC than during her previous birth at an urban facility: “When I had my previous child, I just laid in the bed and they were like, hey, here, take this, go back to sleep. But with them, I did the [birthing] ball, I did water, different stretches. Yeah, I mean, I felt like I was in control.” Respondent 10 reported, “And then I started actually being in labor and they were like, well, you know, sometimes to help with the pain, you can take a shower. I had never done that before, so that was really nice to tell me.” Although experiences with pain management were overwhelmingly positive, Respondent 12 expressed that she had wanted an epidural, did not receive it in time, and had to give birth without an epidural.

Eliciting and respecting patient desires went beyond pain management decisions. Respondent 2 noted, “I was able to deliver him vaginally. That’s what I’ve always wanted with my pregnancies. And I think they really, basically let me do it, let me handle it by myself. And that’s what I wanted.” The providers’ focus on meeting the patients’ needs and wants was clearly expressed by Respondent 13: “Yeah, I think everything was in my control…everybody was working with me on what I wanted to do with my delivery or asking me if everything was OK and providing me with what I needed.”

Perception Changes and Recommendations were mentioned by multiple MCC patients. Numerous patients mentioned having negative perceptions of the rural hospital prior to giving birth at the MCC, and that their perceptions changed after their experiences. Respondent 11 stated: “Prior, this is going to sound bad—but it’s in [name of rural town], and I guess I just didn’t think it was going to be wonderful. But when I took my tour, it definitely changed my mind. It was beautiful inside. It was very clean, very well maintained, all the workers were wonderful, very friendly, very personable, and through my birthing experience and afterward they were amazing.” When comparing her birth experience at the MCC to the hospital where she gave birth previously, Respondent 13 said: “I didn’t, you know, I expected maybe less? But it was like a really nice place to give birth in.” Respondent 4 mentioned that the MCC “was just not anything of like the negative things that I have heard.” Additionally, Respondent 6 mentioned recommending the MCC to others based on her experience:

“I was more than happy…I bragged about it. A lot of people give like a bad rep…you know how they do little bitty hospitals, they think they’re the worst when sometimes they’re not… I didn’t tell many people that I was actually giving birth in [name of rural town], because of the way people think about it, they would be like, ‘why would you do that?’ …And [since the birth] I’ve actually told another girl—I was like, hey, have your baby there, and she had an amazing experience too. And everybody that went after me, I was like, well I had my baby there, great experience.”

Respondent 10 reiterated the importance of the MCC, noting, “…yeah, that hospital, it needs to stay open for women that are low risk, because not a lot of people get treated that well after having a baby. You just feel like a number. So, it’s an amazing hospital.”

### 3.3. Integration and Triangulation of Findings

Data integration and triangulation revealed areas of convergence between quantitative and qualitative findings related to distance to care and perceptions of safety. Quantitative analyses demonstrated substantially shorter distances to care for MCC patients compared with those delivering at the tertiary care hospital, which aligned closely with patient narratives emphasizing proximity as a critical factor shaping birth location decisions, logistical feasibility, and family support. Similarly, low rates of NICU utilization and favorable clinical outcomes at the MCC converged with qualitative accounts describing attentive, responsive care and a sense of safety within the provider model and care environment. Areas of complementarity were evident in patient agency and decision-making: while clinical outcomes such as mode of delivery and epidural use were similar across settings, qualitative findings provided context to illustrate how patients experienced autonomy, felt heard by providers, and actively participated in care decisions at the MCC. In contrast, one area of divergence emerged around neonatal care expectations; despite low NICU utilization and safe transfer protocols for MCC births, some interview respondents expressed a desire for on-site NICU services. Finally, qualitative interviews provided data on how patient perceptions of the rural MCC changed after a positive birthing experience, which was not an area for which relevant quantitative data were available. Together, these integrated findings highlight how comparable clinical outcomes are experienced differently depending on care context, proximity, and relational aspects of maternity care (see Table A1 for joint display of integrated findings).

## 4. Discussion

This mixed-methods study of outcomes associated with opening a rural MCC for low-risk patients found similar labor and delivery outcomes to those of the suburban tertiary care center where patients had delivered prior to its opening, and patients of the rural MCC reported reduced distance to care and high rates of satisfaction. The MCC provided accessible, local maternity services to a community of predominantly low-income, Hispanic families near their homes using a risk- and resource-appropriate care delivery model of mainly family medicine physicians and CNMs. Most births across the MCC and the comparison group were spontaneous vaginal deliveries with similar five-minute Apgar scores and a comparable prevalence of cesarean deliveries, postpartum hemorrhage, low birthweight, and breastfeeding. NICU use was significantly lower in the MCC cohort. Qualitative findings expanded upon the quantitative findings, demonstrating patient satisfaction with the physical environment and positive experiences of supportive providers who offered individualized attention and gave families a sense of agency in their care.

Births at the MCC were primarily attended by family medicine physicians and CNMs. The scope of practice for family fedicine physicians and CNMs includes management of low- to moderate-risk births. We found that within this scope of training and practice at the MCC, family medicine physicians and CNMs managed labor with low rates of morbidity and mortality. Family medicine physicians who provide cesarean sections as primary surgeons disproportionately provide labor and delivery care in rural communities [29], and family medicine physicians and CNMs have been shown to manage low- to moderate-risk deliveries with fewer interventions than obstetricians [30,31,32]. Birth outcomes for individuals with low-risk pregnancies improve with right-sized low-intervention models of care, which yield lower rates of pain medication and cesarean births and higher rates of breastfeeding [33,34]. Our qualitative data on the provider model and hospital care environment complemented quantitative findings, as MCC patients reported personalized care and hands-on management of pain during labor. Supporting family medicine training in cesarean sections and providing training opportunities for both family edicine physicians and CNMs in rural settings may improve access to quality person-centered care.

MCC patients overwhelmingly described positive experiences and a sense of relationship with the attending providers and staff, including a sense of shared care and supportive labor offerings. The importance of facilitating pain management plans is also described in a study by López-Toribo et al. in which postpartum participants described obstacles to shared decision-making from providers and a lack of personal agency during their births, suggesting that the “establishment of mutually respectful relationships between clinicians and women” was the best way to improve participation in shared decision-making [35]. Shared decision-making and empowerment during the perinatal period can improve health outcomes [36], and respectful maternity care is a particularly important concept in communities with inequitable health outcomes [37,38], making the experiences of agency described by our study population particularly evocative.

The opening of the MCC reduced distance to care for rural patients, a finding for which we saw convergence across quantitative and qualitative data. Quantitatively, we found that nearly one-third of MCC patients lived five or fewer miles from the rural hospital, while less than 1% of patients in the comparison cohort resided as close to the suburban center. The anticipation of driving long distances during labor, for the patient and family, and of navigating a large, unknown medical system, can cause increased stress for rural birthing people [39]. Similarly to Blythe et al., patients of the MCC reported relief in the proximity of birth care [40]. This model provides a counter-narrative to current trends in US maternity care where many healthcare systems are closing obstetric units [3] or consolidating maternity care to larger, higher-acuity hospitals staffed by obstetric specialists, providing consultation remotely or intermittently for surrounding rural areas [11,41,42]. This ‘hub and spoke’ model contributes to the framing of rural maternity care as deficient, suggesting that what is available locally, particularly to historically marginalized communities, is insufficient compared to what is available within urban or suburban healthcare institutions [43]. Interview participants voiced this perspective when noting that they anticipated the MCC to be of lower quality because of its rural location. Labor and birth experiences changed patients’ prior perceptions of the MCC, building trust in the local CAH. This shift in attitude is important because it can predict improved engagement with the hospital overall, counteracting medical mistrust, and has the potential to improve long-term health outcomes across the life course [44].

The prevalence of preterm birth and low birthweight infants was similar across the MCC population and the suburban tertiary care center population. However, the 2% prevalence of low birthweight infants born at the MCC is less than one quarter of the 8.6% county-level prevalence, and the 1.5% prevalence of preterm birth at the MCC is much lower than the 12.5% county-level prevalence [45]. This is likely due to guidelines that allow only a medically low-risk subset of the county population to deliver at the MCC, and to the increased accessibility to local health care enhanced by virtual consults with obstetric specialists at the tertiary care center.

One area of divergence between quantitative and qualitative findings emerged around neonatal care expectations. While the lack of a NICU was not a concern for all patients, given the low NICU utilization rate, some MCC patients reported a desire for the hospital to add a NICU in the future. Access to an on-site NICU may not have been a priority for this mostly multiparous and low-risk birthing population, but the high prevalence of NICU use at the suburban tertiary care center for the low-risk comparison cohort in the years prior to the MCC opening may have contributed to a perception that many infants will need NICU services [46,47]. The ready availability of a NICU likely led to higher use and many short-stay observations at the suburban tertiary care center, as hospitals with a NICU may lower their clinical thresholds for NICU use, contributing to higher admission rates [48,49]. While only a small percentage of infants born to low-risk mothers need the high-acuity, high-intervention care that can be the norm in larger hospitals with NICUs, rural maternity units in hospitals without a NICU must maintain readiness for obstetric and neonatal emergencies. A well-functioning rural maternity unit must maintain staff preparedness and stand ready to address obstetric emergencies (e.g., operating rooms, anesthesia, simulation training for maintenance of staff skills, and rapid transfers) [12,50,51]. Obstetric readiness training in rural maternity care is essential both for local access to high-quality birthing care and for addressing the needs of emergency perinatal care [52].

We excluded 10 MCC patients from the quantitative analysis who delivered at the MCC despite not meeting low-risk criteria for admission. Of these 10 patients, three presented at the emergency room with a breech precipitous delivery, three experienced preterm premature rupture of membranes, two were induced for a preterm IUFD, and two were induced after presenting with pre-eclampsia before 37 weeks’ gestation. Four of these excluded deliveries resulted in neonatal deaths (two of these neonates were non-viable). After excluding these 10 patients from our analysis, there were no neonatal or maternal deaths in either the MCC or comparison cohort, and there were two IUFDs in the MCC cohort compared to one at the tertiary care center [53,54]. This study establishes that for risk-appropriate pregnant patients, birth outcomes at a Level 1 MCC are non-inferior to those at an academic tertiary care center. It is important to clearly communicate the admission criteria to community members to ensure risk-appropriate use of Level 1 MCC care, as the opportunity to deliver locally is highly desired by patients and their families. As increased distance to maternity care has been associated with increased risks of negative maternal and infant health outcomes [41], having locally available, risk-appropriate care for pregnant patients is a critical component of efforts to address the national and rural maternity care crisis. This is particularly true for lower-income and minoritized populations, who are more likely to encounter challenges accessing obstetric care [18] and who tend to bear higher risks of negative maternal and infant health outcomes [55].

Given the financial challenges faced by rural hospitals in maintaining labor and delivery services, tailored efforts are needed to support rural hospitals so that they can maintain the provision of high-quality, risk-appropriate, and locally available maternity care in their communities. Rural hospitals have higher rates of underinsured, uninsured, and Medicaid-insured patients, so rural facilities with lower birth volumes generate less revenue for birth care services on average compared to facilities with higher proportions of privately insured patients [56]. The incentives and/or loan repayment programs that are used to attract providers to rural areas require subsidy from state or federal governments or from large health care systems. To improve the accessibility of labor and delivery care in rural communities, health care systems should value rural and regional partnerships beyond models that centralize perinatal care at larger centers, and should provide financial and quality improvement support for low-volume rural facilities working to maintain labor and delivery services [5,57]. For example, enabling low-risk patients to deliver locally and receive risk-appropriate care in their community rather than in a further-away tertiary care center may in turn increase access to specialty care for high-risk patients, a cost-sharing collaborative approach and public health-minded system of healthcare. Our findings buttress the work of the American College of Obstetricians and Gynecologists (ACOG) and the Society for Maternal-Fetal Medicine (SMFM) on levels of maternal care, an integrative system for risk-appropriate perinatal regionalized care [58]. In the 1970s, the US regionalized standards for neonatal care to improve neonatal outcomes without similar organizational efforts for pre-delivery care.. Similar studies evaluating safety and efficacy of regionalized perinatal care systems are needed to positively impact our current maternity crisis.

Our study has several strengths. The use of a mixed-methods framework enabled the triangulation of findings. This mixed-methods design facilitated comparisons of narrative findings with clinical EHR data to gain a richer understanding of outcomes associated with opening and sustaining a rural maternity center for low-risk patients. We conducted an in-depth chart review to ensure consistent EHR data on MCC and comparison cohorts. The group of interviewees was diverse, comprising different racial and ethnic identities and language preferences.

Our findings should also be interpreted within the limitations of the study design. Our quantitative outcomes were limited to diagnosis codes and provider notes available in the EHR system. While MCC and comparison cohorts were drawn from the same prenatal clinics and were similarly low-risk, we cannot exclude the possibility of residual confounding by unmeasured social or clinical factors. Although the comparison cohort preceded the MCC reopening, we do not believe temporal confounding meaningfully biased results. Maternity care practices remained stable across periods, and many MCC clinicians had previously trained or practiced at the comparison hospital, supporting continuity in clinical approaches across sites and time. Participants in the interviews self-selected, only represented a subset of the patient population who gave birth during the first year of the MCC’s operation, and the recruitment technique was a phone call invitation. Thus, participant identities and experiences may not have been representative of the full population or changes in experiences over time. Those who were not accessed via phone recruitment may have included potential participants who did not have access to consistent phone services or who felt negatively toward their experiences at the MCC. Patients in the comparison cohort were not interviewed and may have had similarly positive comments to make about their birthing experiences.

## 5. Conclusions

Rural maternity care is often framed using a deficit model, implying that birth at a larger, higher-resourced institution always provides a better experience; however, we observed similar birth outcomes between this critical access hospital and the larger tertiary care facility among a low-risk patient population, recognizing the possibility of unmeasured confounding. Its small size and the proximity of the rural MCC to the population it served provided patients access to a care team of mostly family medicine physicians and CNMs who created an environment that supported patient autonomy and satisfaction with care [43,59]. While findings may not generalize beyond low-risk populations or rural maternity care centers operating in similar policy and clinical contexts as NC, they provide important evidence of what is possible when rural hospitals are adequately supported to deliver low-risk maternity care. Further, local care has the potential to alter negative community perceptions about the quality of care provided in local, rural hospitals, as demonstrated in this population of predominantly low-income Hispanic patients who expressed a sense of agency and a feeling of being seen and personally supported during their birth. Health literacy and health insurance are factors when patients choose their site of delivery. Provision of maternal care in a rural community can enhance health literacy and instill confidence in the local care through patient education, which builds familiarity with the services provided. This community education may reduce the practice of bypassing closer birthing facilities in favor of more urban locations, which is a behavior associated with White, non-Hispanic race/ethnicity and private insurance [60]. A local MCC, in a rural county with a large population of low-income and Hispanic families, is likely to make a difference for care access and, with time, an established reputation may attract many patients, which will help with long-term financial viability of the MCC. In the meantime, the availability of local, high-quality, and risk-appropriate maternity care directly addresses some of the healthcare inequities reflected through the maternity care crisis in the US that has disproportionately affected rural communities.

Family medicine physicians are cost-effective, given their generalist training and lower salaries compared to obstetricians, and in partnership with CNMs, these physicians can provide safe, effective maternity care for low-risk populations. Such staffing models must be a policy priority with financial investments that sustain the model for rural hospitals. This includes state Medicaid expansion, given the dependency of rural hospitals on these funds. Expansion of the federal Health Resources & Services Administration (HRSA) Primary Care Training and Enhancement grants, which include programs that provide maternal health training in rural settings, can address some rural labor and delivery workforce issues. New state programs are similarly supporting training in rural areas, knowing that clinicians and staff are more likely to stay in rural areas if they train there. Medical and nursing school admission policies that prioritize students from rural areas may also help to address workforce needs. Including smaller rural hospitals in larger health care systems can buffer the finances needed to support rural hospitals’ ability to maintain safe and high-quality labor and delivery services. Research can clarify the ongoing costs for larger health systems in supporting affiliated rural hospitals, including the benefits of decompressing volume at larger tertiary care centers, teaching and training opportunities, and the level of public and government support needed to sustainably address the rural maternity care crisis.

## Figures and Tables

**Table 1:** Descriptive Characteristics of the Maternity Care Center (MCC) and Comparison Suburban Tertiary Care Center Cohorts.

Variable	Hospital				
	MCC (*n* = 402)		Comparison (*n* = 593)		
	*n*	(%)	*n*	(%)	Chi-Square *p*-Value
Race/ethnicity					0.08
Hispanic/Latino	279	(69.4)	444	(74.9)	
Non-Hispanic White	68	(16.9)	72	(12.1)	
Non-Hispanic Black	44	(10.9)	51	(8.6)	
Non-Hispanic Other	10	(2.5)	25	(4.2)	
Asian	1	(0.2)	1	(0.2)	
Preferred language					0.06
English	232	(57.7)	295	(49.8)	
Spanish	169	(42.0)	292	(49.2)	
Arabic	1	(0.2)	1	(0.2)	
Sign language	0	0	2	(0.3)	
Missing	0	0	3	(0.5)	
Insurance at Delivery					**<0.001**
Medicaid	276	(68.7)	550	(92.8)	
Private	59	(14.7)	31	(5.2)	
Uninsured	67	(16.7)	12	(2.0)	
Maternal age					0.91
16–20	50	(12.4)	71	(12.0)	
21–25	123	(30.6)	175	(29.5)	
26–30	102	(25.4)	141	(23.8)	
31–35	80	(19.9)	122	(20.6)	
36–40	38	(9.5)	68	(11.5)	
41–45	9	(2.2)	16	(2.7)	
Parity					0.96
Multipara	259	(64.4)	387	(65.3)	
First birth	128	(31.8)	184	(31.0)	
Grand multipara (>4 births)	15	(3.7)	22	(3.7)	
History of preterm birth (<37 completed weeks gestation)	38	(9.5)	50	(8.4)	0.58

Bold text indicates statistically significant differences at (*p* < 0.05).

**Table 2:** Labor and Delivery Outcomes for the Maternity Care Center (MCC) and Suburban Tertiary Care Cohorts.

Variable	Hospital					
	MCC (N = 392)		Comparison (N = 593)			
	N	(%)	N	(%)	OR (95% CI)	Wald Chi-Square *p*-Value
**Type of labor**						
Spontaneous	245	(62.5)	384	(64.8)	(ref)	
Induced	125	(31.9)	163	(27.5)	0.8 (0.6, 1.1)	0.20
Scheduled Cesarean Section	22	(5.6)	46	(7.8)	1.3 (0.8, 2.3)	0.29
**Delivery Service Type**						
Obstetrics/Gynecology	6	(1.5)	499	(84.2)	(ref)	
Family Medicine	267	(68.1)	72	(12.1)	**0.003 (0.0, 0.01)**	**<0.0001**
Certified Nurse Midwifery	119	(30.4)	22	(3.7)	**0.002 (0.0, 0.01)**	**<0.0001**
**Delivery type**						
Vaginal	340	(86.7)	502	(84.7)	(ref)	
First Cesarean Section	33	(8.4)	54	(9.1)	1.1 (0.7, 1.7)	0.66
Repeat Cesarean Section	19	(4.9)	37	(6.2)	1.3 (0.7, 2.3)	0.34
**Epidural use (vaginal only) ^a^**						
No	146	(42.9)	204	(40.6)	(ref)	
Yes	194	(57.1)	298	(59.4)	1.1 (0.8, 1.5)	0.51
**Birth outcome**						
Live birth	390	(99.5)	592	(99.8)	(ref)	
Intrauterine fetal demise	2	(0.5)	1	(0.2)	0.3 (0.0, 3.6)	0.37
**Postpartum hemorrhage > 1000 mL**						
No	362	(92.4)	563	(94.9)	(ref)	
Yes	30	(7.7)	30	(5.1)	0.6 (0.4, 1.1)	0.10
**Gestational age at birth**						
Term (≥37 weeks)	386	(98.5)	575	(97.0)	(ref)	
Preterm (<37 weeks)	6	(1.5)	18	(3.0)	2.0 (0.8, 5.1)	0.14
**Birthweight**						
Low (<2500 g)	8	(2.0)	13	(2.2)	1.1 (0.5, 2.7)	0.83
Normal (2500 to <4000 g)	359	(91.6)	528	(89.0)	(ref)	
Large for gestational age (≥4000 g)	25	(6.4)	52	(8.8)	1.4 (0.9, 2.3)	0.17
**Apgar score at five minutes**	Mean	SD	Mean	SD		*t*-test
	8.8	(0.66)	8.9	(0.56)		*p* = 0.22
**Neonatal intensive care unit stay ^b^**						
No	382	(97.5)	394	(66.4)	(ref)	
Yes	10	(2.5)	199	(33.6)	**19.3 (10.1, 37.0)**	**<0.0001**
**Infant Feeding Status at Discharge**						
Any breastfeeding	353	(90.1)	552	(93.2)	(ref)	
Only formula feeding	37	(9.4)	40	(6.8)	0.7 (0.4, 1.1)	0.12
N/A	2	(0.5)	1	(0.1)	**--**	

^a^ Epidural use calculated among vaginal deliveries only. ^b^ This required a transfer to the nearest NICU for MCC deliveries, while this occurred at the suburban tertiary care center for comparison cohort deliveries. Bold text indicates statistically significant differences at (*p* < 0.05).

## Data Availability

The de-identified dataset that underlies the findings described in this article is not publicly archived because of participant privacy concerns and ethical restrictions. However, data may be available from the corresponding author upon reasonable request and contingent upon approval of a data-sharing agreement and relevant institutional review. Interested researchers should contact Kathryn Wouk at kwouk@pire.org to discuss access conditions.

## Abbreviations

The following abbreviations are used in this manuscript:

ACOG	American College of Obstetricians and Gynecologists
BMI	Body Mass Index
DM	Diabetes mellitus
HTN	Hypertension
IUFD	Intrauterine Fetal Demise
MCC	Maternity Care Center
NICU	Neonatal Intensive Care Unit
SMFM	Society of Maternal-Fetal Medicine
TOLAC	Trial of labor after cesarean

## Appendix A

**Table A1:** Joint Display of Integrated Findings.

Qualitative Data	Themes	Convergent Quantitative Data
“I had my baby within 15 min of arriving at the hospital. So, I think that if [the MCC] had not been there, I would have had my baby in a car.” “I did not want to consider traveling the 45 min it normally took me to get to the other location.” “I have a child that suffers from [medical condition], and I didn’t want to go very far to the other hospital…That was the most important thing, to not go so far away because of my son.” “So, my husband and I spoke and one of the advantages that we looked at the most was that it was here near the house and since we have two; I have a little girl of seven years old. He told me, we are close, I can come and go from the hospital to see the girl. And that was also what motivated us the most in the child being born here.” “And, yeah, since my pregnancy wasn’t a high risk, you know, I would just go to a closer place to where I live.” “It was really close to my house and we were able to get there like fairly quick.” “She asked me which hospital I preferred and I told her the closest one.”	Distance to care	32% of MCC patients lived 5 or fewer miles from the hospital, while less than 1% of patients resided 5 or fewer miles from the tertiary care hospital. The median distance to care was 16 miles for the MCC cohort and 27 miles for the comparison cohort who delivered at the tertiary care hospital.
“I’d seen [doctor’s name] the whole time I was there. I mean, usually you see the doctor one time, and you never see him again.” “I would say, the lady, the person who helped me deliver—I wish I knew her title, I want to call her midwife, but she may not even be a midwife—but she was very encouraging.” “She immediately ran to get the doctor that helped me deliver my baby. She immediately went and it wasn’t even like the doctor that was there. I think it was a midwife. She immediately went to get her. And I had my baby like five minutes later…But the lady, the midwife that helped me deliver, was very, very good. And I just remember thanking the nurse for for only having to tell her once that I, that this is what I’m feeling, like I can feel him coming. And she immediately responded. She didn’t say, Mom, don’t push, or nothing. She just went ahead, and you know, I felt heard. That’s what I told them after giving birth.” “Like I couldn’t have had a better support system there with the nurses and the doctor and the midwives, even from whenever I, before I called and went in, I called and the midwife talked to me and she coached me while I was at home.” “I mean, anything to do to help [the MCC] for their maternity ward, I would do it, just so they can end up getting the NICU for mothers like me who need it.” “I really don’t have any complaints. In the future, I would like to see them have a NICU, just in case. But other than that, they were wonderful.”	Provider model and hospital care environment	Deliveries at the MCC were significantly more likely to be attended by a family medicine physician (68.1%) or certified nurse-midwife (30.4%) compared to those at the tertiary care hospital. Infants at the tertiary care hospital were significantly more likely to experience a NICU stay after delivery (33.6%) compared with infants at the MCC (2.5%) who were transferred as necessary to the closest NICU (*p* < 0.0001) shortly after birth.
“Yeah, everything went as planned because I was able to deliver him vaginally. That’s what I’ve always wanted with my pregnancies. And I think they really basically let me do it, let me handle it by myself. And that’s what I wanted.” “They were very like, whatever you want—if you want medicine, that’s fine, if you don’t, then that’s fine, too. We’ll talk you through all of it. Never pushy on either one, which was fantastic because sometimes I felt that way at the other place.” “And they never, they never really asked about medicine. My husband would say, hey, like if she wanted to get that epidural now could she? And they were like, absolutely, she just needs to tell us.” “Well, I was in a little pain that day, but the girl was already 6 cm dilated and there they asked me if I wanted something for the pain. But I didn’t. I didn’t choose anything because a lot of people tell me it’s bad to choose it, something they give you so it doesn’t hurt. I preferred to have it that way, natural.” “There was no pain management or anything provided for me.”	Patient agency/Decision-making	86.7% of patients at the MCC had vaginal deliveries compared to 84.7% of patients in the comparison cohort who delivered at the tertiary care hospital. 57.1% of patients at the MCC who had vaginal deliveries used an epidural, compared to 59.4% of patients who delivered vaginally at the tertiary care hospital.
“And it was… I didn’t, you know, I expected maybe less? But it was like a really nice place to give birth in.” “Prior, this is going to sound bad—but it’s in [name of rural town], and I guess I just didn’t think it was going to be wonderful. But when I took my tour, it definitely changed my mind. It was beautiful inside. It was very clean, very well maintained, all the workers were wonderful, very friendly, very personable, and through my birthing experience and afterward they were amazing.” “[The MCC] was just not anything of like the negative things that I have heard.” “I was more than happy…I bragged about it. A lot of people give like a bad rep…you know how they do little bitty hospitals, they think they’re the worst when sometimes they’re not… I didn’t tell many people that I was actually giving birth in [name of rural town], because of the way people think about it, they would be like, ‘why would you do that?’ …And [since the birth] I’ve actually told another girl—I was like, hey, have your baby there, and she had an amazing experience too. And everybody that went after me, I was like, well I had my baby there, great experience.” “…yeah, that hospital, it needs to stay open for women that are low risk, because not a lot of people get treated that well after having a baby. You just feel like a number. So, it’s an amazing hospital.”	Perception changes and recommendations	N/A—no relevant quantitative data was collected on this theme.
